# Intravitreal injection of β-crystallin B2 improves retinal ganglion cell survival in an experimental animal model of glaucoma

**DOI:** 10.1371/journal.pone.0175451

**Published:** 2017-04-06

**Authors:** Fabian Anders, Julia Teister, Aiwei Liu, Sebastian Funke, Franz H. Grus, Solon Thanos, Harald D. von Pein, Norbert Pfeiffer, Verena Prokosch

**Affiliations:** 1 Experimental and Translational Ophthalmology, Department of Ophthalmology, University Medical Center of the Johannes Gutenberg-University Mainz, Mainz, Germany; 2 Institute for Experimental Ophthalmology, School of Medicine, Westfalian-Wilhelms-University Münster, Münster, Germany; 3 Institute of Neuropathology, University Medical Center of the Johannes Gutenberg-University Mainz, Mainz, Germany; Schepens Eye Research Institute, Massachusetts Eye & Ear, Harvard Medical School, UNITED STATES

## Abstract

Purpose of this study was to investigate firstly specific proteomic changes within the retina in the course of an animal glaucoma model and to identify secondly new approaches for neuroprotective, therapeutic options in glaucoma by addressing those specific changes. Intraocular pressure was elevated through cauterization of episcleral veins in adult Sprague Dawley rats. Molecular and morphological changes were surveyed using mass spectrometry, optical coherence tomography as well as immunohistochemical cross section- and flat mount stainings. By quantifying more than 1500 retinal proteins, it was found that the HspB5 protein and numerous beta-crystallins showed a uniform and unique shifting expression pattern as a result of different periods of elevated IOP exposure. Crystallins showed a significant downregulation (p<0.05) after 3 weeks of elevated IOP and an upregulation after 7 weeks. Counteracting those typical changes, an intravitreal injection of β-crystallin B2 at the time of IOP elevation was found to reduce retinal ganglion cell loss (p<0.05), decrease of the retinal nerve fiber layer (p<0.05) and impairment of the optic nerve. Ultimately, proteomic data revealed that β-crystallin B2 might influence calcium-depended cell signaling pathways with severe effect on apoptosis and gene regulation. In this context especially annexin A5, calcium-transporting ATPase 1 and various histone proteins seem to play a major role.

## Introduction

Glaucoma is the major cause of blindness worldwide [1], characterized by progressive retinal ganglion cell (RGC) loss leading to irreversible visual field defects. Elevated intraocular pressure (IOP) is a major risk factor of glaucoma and lowering of IOP remains the mainstay of glaucoma treatment. However, RGC loss might proceed despite successful IOP reductions [2, 3]. Thus the underlying mechanisms of RGC loss remain obscure [4]. Elevated IOP leads to various molecular changes within the retina and optic nerve head, potentially initiating a secondary self-propagating process of RGC degeneration [5–23].

Besides the better understanding of the disease, there is a high need for neuroprotective drugs, which support the conventional medical treatment by influencing the consistency of the neuronal network, particularly the RGCs [24]. One interesting group of molecules in this context are the crystallins.

Glaucomatous cell death is commonly associated with inflammatory and metabolic processes. The protein family of crystallins comprise of three major subgroups: alpha, beta and gamma crystallins. Those proteins went within a relatively short period of time from being considered lens-specific proteins to be recognized as retinal proteins as well, in particular in the retinal ganglion cells [25, 26]. Heat shock proteins (HSP) are a group of inducible proteins, regulated for example upon central nerve system injury. Particular regulations of HSPs and small heat shock proteins (HspB) have already been observed in context with several neurodegenerative diseases [27, 28]. Besides this, increased antibody levels against different kinds of HSPs in aqueous humor of human glaucoma patients could be verified in a multivariate experimental approach [29]. Especially several experimental studies indicated a neuroprotective potential of β-crystallin B2 when it comes to neuronal impairment *in vivo* and *in vitro* [30–32]. Concerning the crystallin function, a conclusive mode of action could not be revealed yet [26, 33, 34]. Thus, crystallins might function as critical modulators in the course of glaucoma and might be integral to glaucomatous neurodegeneration.

The purpose of this study was the identification of retinal proteins specifically altered in the course of glaucoma in an experimental animal model and the review of these alterations regarding their neuroprotective/neurotoxic influence. As it will be shown, intervention in the typical crystallin protein expression pattern leads to distinct neuroprotective effects with respect to RGC survival in the represented glaucoma animal model.

## Material and methods

### Ethical statement and animals

All experiments were conducted in accordance with the Association of Research in Vision and Ophthalmology Statement for the Use of Animals in Ophthalmic and Vision Research. The committee for animal research specifically approved this study (Landesuntersuchungsamt Rheinland-Pfalz), permission-no: 14-1-085). Animals were housed in standard accommodations provided by the translational animal research center (TARC) of the Johannes Gutenberg University of Mainz with food and water provided ad libitum and a 12-h day-night cycle. Surgical procedures were performed exclusively on the left eye of 250–270 g weighing Sprague Dawley rats under general anesthesia using 0.05 mL medetomidine hydrochloride (Dorbene vet., Pfizer), administered intramuscularly. Additionally, animals were treated topically with oxybuprocain eyedrops (Novesine, OmniVision), before surgery. Further, all water tanks of treated animals were blended with four drops of 500 mg/mL novaminsulfon (Novalgin, Ratiopharm) postoperatively for a period of two days to ensure analgesia. The animals’ health and behavior were monitored postoperatively directly after awaking, the next day and subsequently on a weekly base from laboratory employees with respect to condition of the eyes, quality of defecation, fur, claws and oral cavity. Also animals were checked by skilled stockman in the animal facility on daily base, according to the Protection of Animals Act. Follow-up investigations on animals lasted up to nine weeks after cauterization. In total, 23 animals were included in this study, divided into four experimental groups.

### Induction of elevated IOP and IOP measurement

IOP was elevated through thermic cauterization of three episcleral veins as shown by Shareef et al. [16]. These veins are responsible for the venous outflow and travel close to the sclera from the limbus backwards and anastomose at the equator of the eye. Exposure of the sclera was performed by careful incision of the conjunctiva. Episcleral veins usually form five major trunks almost equidistant around the circumference of the globe. By cauterization of three trunks, venous outflow can be reduced up to 50%, leading to a distinct increase of IOP after two to three weeks [13]. Special care was taken not to damage any surrounding scleral tissue during the surgery. IOP measurements were taken before surgery as baseline values and weekly after cauterization between 9.00 a.m. and 12.00 p.m. with a TonoLab (iCare). Animals were measured awake without general anesthesia. Ten TonoLab readings were taken directly from the display of the instrument for each eye measurement, recorded, and averaged. The IOP appeared increased two weeks after cauterization and remained elevated for the entire length of the study. Animals, which showed no IOP elevation or a return of the IOP to normal levels, were excluded from the study. None of the animals showed an enlarged globe or oedematous cornea or signs of retinal ischemia.

### Intravitreal injection of recombinant β-crystallin B2

β-crystallin B2 was injected intravitreally into the left eye (OS) eyes 3 weeks after cauterization, with beginning of IOP elevation. The protein was expressed heterologous in *e*.*coli* and purified with a HPLC system. Per animal, a single injection of 10 μg β-crystallin B2 in a volume of 3 μL at nasal orientation was conducted. The dose of the β-crystallin B2 injection was chosen according to the work of Böhm et al. [30]. Care was taken not to injure the lens.

### Quantification of retinal ganglion cells

3 and 7 weeks after elevation of IOP, animals were sacrificed under CO_2_ atmosphere, retinas were isolated, flatmounted and immunohistochemistry on retinal flatmounts performed. Briefly, the tissue was fixed in 4% formaldehyde (Carl Roth) at 4°C for 30 minutes and stored overnight in 30% sucrose-solution in PBS, pH 7.4 at 4°C. Then the specimens were transferred in N-methyl-butane (Merck Millipore), which was cooled down in liquid nitrogen, as a final fixation and stored at -80°C until the immunohistochemical processing. The primary anti-BRN3A antibody (Santa Cruz Biotechnology) was diluted in 10% fetal calf serum and incubated overnight at 4° C. After washing in PBS (2 times, 10 min each) and blocking with 0.5% Triton-X (Sigma Aldrich) containing PBS, the incubation of the secondary antibody occurred in for 2 hours at 4°C. Per quarter of explanted retinal tissue, 15 images were taken using a 20x magnification and visualized with a fluorescence microscope (Axiophot Carl Zeiss). RGC quantification was performed automatically with an ImageJ macro (National Institutes of Health, Bethesda, MD) and reviewed manually. For quantifying the RGCs each quarter was oriented to the site of the optic nerve head to ensure that for each individual retina the same number of peripheral and central orientated pictures was taken. The number of RGCs per square millimeter was determined and averaged for each piece of retinal tissue and further averaged per experimental group.

### Optical coherence tomography (OCT) scanning

The retinal nerve fiber layer thickness (RNFLT) was surveyed by an SD-OCT scan using a Spectralis OCT (Heidelberg Engineering). Several adjustments had to be enabled in order to use this device for measurements with rodents. The corneal radius was fixed to 7.7 mm, focus and reference arm were adjusted individually for each animal, but were fixed for the particular animal in follow-up OCT investigations. Scans were recorded using the circular scan option with the optic nerve head as the center and 100 frames per scan. Scans were taken before cauterization and after 3 and 7 weeks of elevated IOP, respectively. Furthermore, OCT investigations were used to observe potential changes in the retinal tissue like ischemia or retinal detachment. Nevertheless, nothing of the kind could be seen.

### Optic nerve axon damage and density

After passing of the animals the parts of the optic nerve with a distance of 5 mm to the optic chiasma belonging to the cauterized eye and the contralateral eye were fixed in 3% glutaraldehyde (Merck) in 0.1 M sodium cacodylate buffer (Serva). Thereafter the samples were embedded in epoxy-resin and semi-thin transverse sections of the optic nerves were cut at 0.65 μm with a glass knife, using an ultramicrotome (Leica). For staining of the optic nerve paraphenylenediamine (Merck) was used. From each optic nerve, up to 6 slices were laid off on an object slide. From the most preserved slice, 20 images were taken using a 100x magnification. All images were ranked after their level of axonal impairment based on the model of Kuehn et al. [35].

### Proteomic analysis

For proteomic analysis, retinal tissue from the rodents was explanted and dissociated using liquid nitrogen and a mortar. Samples from different eyes and animals were collected and treated separately with lysis buffer containing 0.5% n-Dodecyl β-D-maltoside (Sigma Aldrich). Furthermore, the tissue was processed with an ultrasonic bath and ultrasonic wand for efficient cell breakdown. After multiple centrifugation steps, protein concentration was determined by BCA Pierce Protein Assay kit (Thermo Fisher Scientific). To separate the proteins and facilitate a downstream MS analysis, 80 μg of protein lysate per individual retina sample was added to one gel lane of a NuPage Novex 12% Bis-Tris Protein Gel (Invitrogen) and SDS-PAGE was performed accordingly. Every gel lane was cut into 17 pieces, destained and digested with sequencing grade modified trypsin (Promega) over night. C-18 ZipTips (Merck Millipore) were used to clean the samples and separate the peptides from salts and other byproducts. Clean samples were stored at -80°C until the mass spectrometric analysis.

### Protein quantification

Peptides from the trypsin in-gel digestion were analyzed with a capillary LC-ESI-MS system consisting of a C-18 precolumn and a C-18 analytical column to ensure a high resolution during MS measurement. As solvent delivery system a Rheos Allegro HPLC Pump was used with a 50 minute linear gradient system containing water, acetonitrile, methanol and formic acid. Mass spectra were obtained using a LTQ OrbitrapXL (Thermo Scientific). The full-scan mass spectra (m/z 300–2000) were acquired with a resolution of 30.000. Subsequently, mass spectra were utilized in terms of protein identification and quantification using Maxquant (Max-Planck-Gesellschaft). Fixed protein modifications were set to oxidation and acetylation. The match tolerance in mass precision for MS/ MS was adjusted to 20 ppm and 0.5 Da. The false discovery rate for proteins and peptides were set at 0.01, the minimum peptide length was 6 amino acids. Only unique peptide sequences were used for the quantification process. Protein fold-changes were calculated through dividing averaged LFQ intensities from one experimental group by averaged LFQ intensities of the control group and plotted with a logarithmic scale. In this procedure the contralateral eyes within the same experimental group served as a control in order to calculate the deviation. The mass spectrometry proteomics data have been deposited to the ProteomeXchange Consortium via the PRIDE [36] partner repository with the dataset identifier PXD005258.

### Immunohistochemical staining of β-crystallin B2 in retinal cross-sections

Explanted retinal tissue was fixed in 4% formaldehyde for 30 minutes and embedded in paraffin using a standard alcohol / xylene dilution series. The paraffin block was sectioned at 10 μm intervals with a microtome (Leica Reichert Jung). Per individual retina, 10 cross-sections were manufactured and placed on standard object slides. For the staining process itself, the paraffin was removed by storing the object slides at 60°C over night, followed by 10-minute incubation in xylene and an ethanol dilution series (100%, 96% and 70%, each 3 minutes). To ensure the accessibility of the targets, all slides were treated with target retrieval solution (DAKO) for 50 minutes at 70°C. After 1 hour of blocking, the anti- β-crystallin B2 antibody (Santa Cruz Biotechnology) was incubated overnight at 4°C and visualized with a donkey anti-goat TRITC antibody (Thermo Fisher Scientific) through incubation for 2 hours at room temperature. The stained tissue was covered up with DAPI containing mouting medium (Vectashield). In order to compare the intensities, all cross-sections were recorded with the same microscope (Axiophot Carl Zeiss) and identical exposure times (1 second, 50% reinforcement).

### Experimental design

The whole study comprised of 23 Sprague Dawley animals, matched in age and weight. All rodents got a thermic cauterization in order to elevate their IOP on the left eye. For each animal the right eye served as a contralateral control. The animals were further divided into four experimental groups to monitor the effect of elevated IOP in different time states and the effect of an intravitreal injection of β-crystallin B2 on morphological and molecular levels (Table 1).

**Table 1:** Experimental design of the study. The whole study comprised of 23 Sprague Dawley animals, matched in age and weight. All rodents got a thermic cauterization in order to elevate their IOP on the left eye. For each animal the right eye served as a contralateral control.

Exp. Group	Animal number	IOP elevation (OS)	Crystallin injection	Sacrificed after stated week of elevated IOP
Time point 1	8	**+**	**-**	3
Time point 2	7	**+**	**-**	7
β-crystallin B2 1	4	**+**	**+**	7
β-crystallin B2 2	4	**+**	**+**	3

### Statistics and graphical display

All graphs and statistical measurements were designed and calculated with GraphPad Prism v. 6. All experimental data was checked for Gaussian distribution. If Gaussian distribution was given, the statistical significance was calculated with a grouped parametric t-test. If no Gaussian distribution was existent, statistical significance was tested using the non-parametric Mann-Whitney U test. In both cases a p-value of less than 0.05 was considered as statistically significant. All indications given on n for the respective figures represent the number of animals used for the specific dataset.

## Results

### Effect of elevated intraocular pressure on retinal morphology

**Fig 1:**
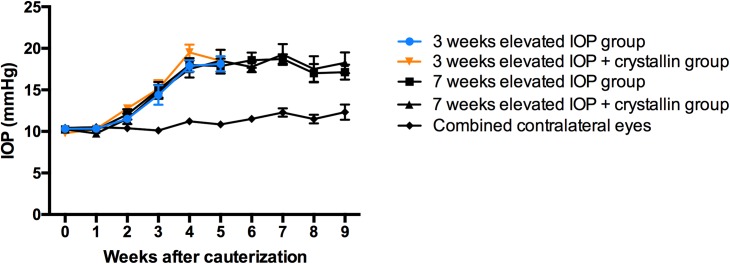
IOP monitoring after episcleral vein cauterization. IOP comparison from eyes treated by thermic cauterization in four different experimental groups (cf. individual n in Table 1) and their combined contralateral controls (n = 23). IOP elevation can be observed in about 3 weeks after surgery and resulted in an average increase up to 17.40±0.78 mmHg, while the contralateral eye remains at baseline level (p<0.0001, parametric t-test, ±SEM).

Thermic cauterization of three episcleral veins induced a significant increase of the IOP in the animals’ eyes (combined 17.40±0.78 mmHg) for the whole period of follow-up compared to baseline levels (10.15±0.13 mmHg) and contralateral fellow eyes (11.25±0.24 mmHg) (Fig 1).

**Fig 2:**
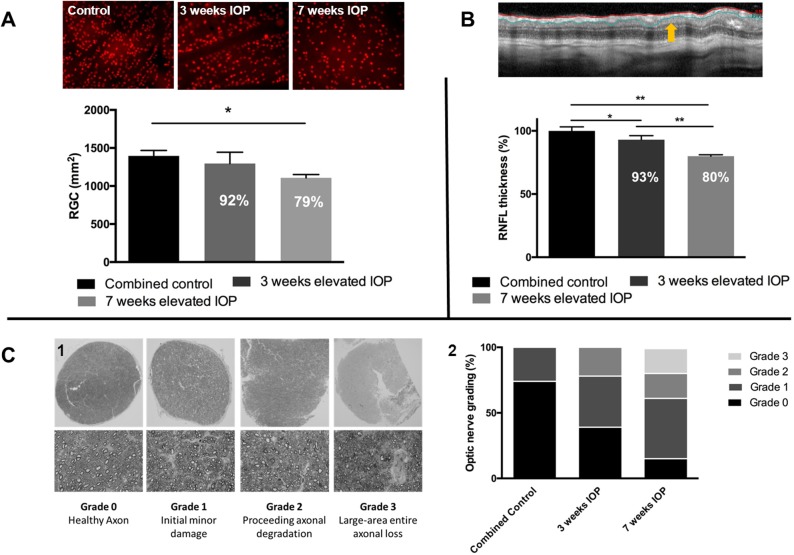
Effect of elevated intraocular pressure on retinal morphology. Brn3A immunohistological staining showed a decrease of RGC due to elevated IOP (A). Compared to the combined control retina (n = 10), which was not exposed to elevated IOP, the 3 weeks (n = 5) and 7 weeks elevated IOP group (n = 5) show a RGC loss of 8% respectively 21% (*p<0.05, parametric t-test, ±SEM). The RNFLT was measured with a 12° diameter circular B-scan, decrease of RNFLT (B) was comparable to RGC loss (*p<0.05, **p<0.01, parametric t-test, ±SEM, n = 5). Grading of the optic nerve (C1) allowed a rough classification of IOP exposed cross-sections. Evaluation of the data showed clear impairment of the optic nerve with progressional period of elevated IOP (n = 7) (C2).

Elevation of IOP resulted in a continuous progressive RGC loss, decrease in retinal nerve fiber layer thickness as well as an impairment of the optic nerve over the course of time. Quantification of RGCs through staining of Brn3a^+^ cells was chosen to pass on a staining with FluoroGold (FG), which would have required another invasive intervention for the animals. Furthermore, it was shown that Brn3a staining leads to similar results compared to FG staining in the retina [37]. In retinal tissue exposed to elevated IOP, the RGC number was 8% lower after three weeks (1295±148.7 RGC/mm^2^) and 21% lower after 7 weeks (1108±43.6 RGC/mm^2^, p<0.05) of IOP elevation, compared to the combined contralateral control (1396±72 RGC/mm^2^) (Fig 2A). The thickness of the retinal nerve fiber layer decreased in a similar level compared to the RGC loss. After three weeks of IOP elevation a decrease of 7±3,37% could be observed, after seven weeks the decline was 20±3,03% (p<0.01) (Fig 2B). In cross sections of the optic nerve a progressive degradation of single axons could be seen. Optic nerves of contralateral eyes, showed almost exclusively healthy axons and partly minor damages, while the axonal impairment was distinctly higher with increasing length of IOP exposure (Fig 2C).

### Proteomic changes in the course of IOP elevation

**Fig 3:**
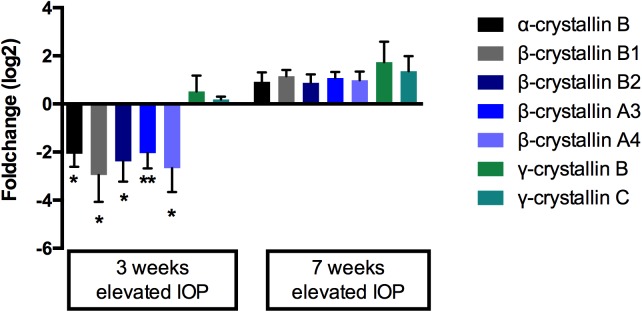
Regulatory changes of crystallin proteins on elevated IOP. Analyzed members of the different crystallin subclasses showed a unique expression profile at both investigated timepoints of IOP elevation (t_1_ n = 8, t_2_ n = 7). (*p<0.05, **p<0.01, Mann-Whitney U test, ±SEM). Fold-changes are plotted using a logarithmic scale with a basis of 2.

The mass-spectrometric profiling revealed regulatory changes in the expression of crystallin proteins. Interestingly, all α- and β-crystallin proteins were found to be down-regulated after three weeks of elevated IOP and up-regulated after seven weeks of IOP elevation (Fig 3).

**Fig 4:**
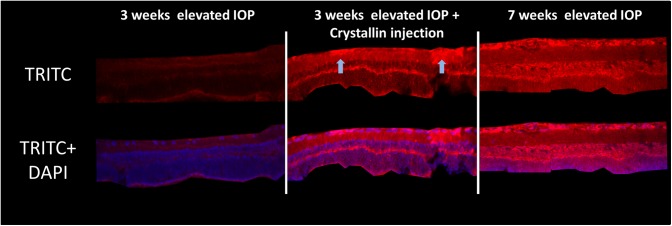
β-crystallin B2 staining in retinal cross-sections. IHC staining could validate the results from mass-spectrometric featured proteomics analyses. After 3 weeks of IOP elevation, almost no β-crystallin B2 could be detected in the retina cross section (n = 3), while a strong increase of protein level could be verified after 7 weeks of elevated IOP (n = 3). Injection of β-crystallin B2 seems to change the protein level in retinal cells after 3 weeks of IOP elevation considerably due to cellular uptake of the crystallin, predominantly by the RGC layer (n = 4).

Hereof, β-crystallin B2 was down-regulated with a fold-change of 0.19 (p<0.05, log_2_ = -2.4) at the early timepoint 1 and up-regulated with a fold-change of 1.8 (log_2_ = 0.85) at the later timepoint 2. The mass-spectrometric results for β-crystallin B2 could further be validated with immunohistochemical staining against β-crystallin B2 in retinal cross-sections. The staining showed the clear increase of β-crystallin B2 in the retinal tissues, which were exposed to 7 weeks of elevated IOP, compared to the 3 weeks’ group. Furthermore, staining of the retinal tissue, which was exposed to elevated IOP for only 3 weeks, but received an intravitreal injection of β-crystallin B2, showed considerable indication of cellular uptake of the injected β-crystallin B2, especially within the RGC layer (Fig 4).

On the contrary, γ-crystallin B and γ-crystallin C did not follow this expression pattern and showed a slight up-regulation at the first time point and increasing up-regulation at time point 2 (Fig 3).

Besides the protein family of crystallins, the majority of all identified retinal proteins showed no noticeable regulation. Exemplary, proteins like Glyceraldehyde 3-phosphate dehydrogenase (GAPDH), 14-3-3 proteins or Tubulin beta-4B can be stated in this context. Glial fibrillary acidic protein (GFAP), however, was found strongly increased in the eyes exposed to elevated IOP, which is certainly induced through the glial response in the course of IOP elevation [38]. An increase of GFAP in a similar study was already shown by Rogers et al., which can be regarded as a further validation of the here presented MS data [39].

### Effect of β-crystallin B2 injection prior to IOP elevation

**Fig 5:**
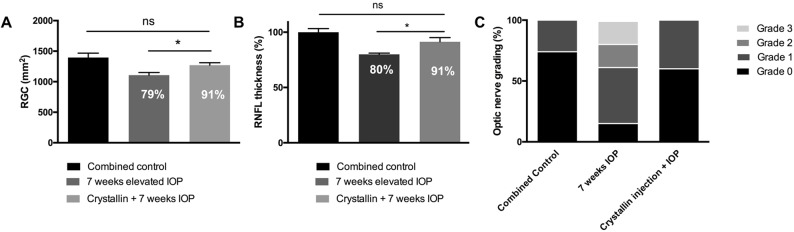
Morphological effect of β-crystallin B2 injection prior to IOP elevation. Injection of β-crystallin B2 results in a 12% higher RGC survival rate (n = 4), in case of IOP elevation, compared to the 7 weeks IOP group (n = 7) (A, *p<0.05, parametric t-test, ±SEM). Similar results could be observed for the decrease of the RNFLT, which was found to be 11% lower (n = 4) than in the IOP group, respectivelly (n = 5) (B, *p<0.05, parametric t-test, ±SEM). Neuroprotective effects could also be analyzed in the optic nerve, where cross-sections of crystallin injected animals (n = 4) showed similar results with respect to the untreated control group (n = 10) (C).

Injection of β-crystallin B2 prior to IOP elevation resulted in considerably increased survival of RGCs. Compared to the seven weeks IOP group, crystallin injected eyes denote 12% (1271±40.8 RGC/mm^2^) less loss of RGCs (Fig 5A), 11±4.5% less decrease in retinal nerve fiber layer thickness (Fig 5B) and considerable healthier axons in optic nerve cross sections (Fig 5C).

### Molecular changes due to crystallin injection

**Fig 6:**
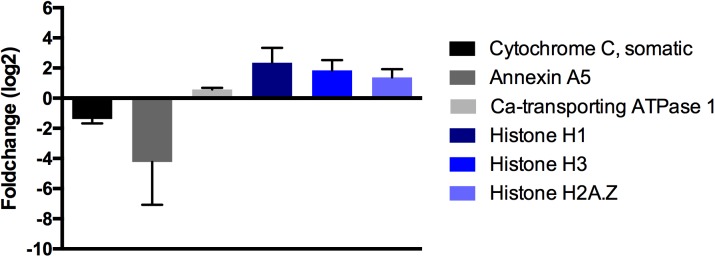
Alterations of protein expression by β-crystallin B2 injection. Crystallin injected animals show proteomic changes in specific calcium signaling key proteins as well as gene regulation proteins (±SEM, n = 4). Fold-changes are plotted using a logarithmic scale with a basis of 2.

Quantified and normalized LFQ intensities of crystallin injected eyes and the seven weeks elevated IOP group, indicate clear evidence for molecular alterations in cellular calcium signaling. Somatic cytochrome C (FC = 0.38, log_2_ = -1.37) and annexin A5 (FC = 0.053, log_2_ = -4.23) were found explicitly down-regulated. Plasma membrane calcium-transporting ATPase 1 was found up-regulated (FC = 1.5, log_2_ = 0.58) as well as histone H1 (FC = 5.1, log_2_ = 2.35), histone H3 (FC = 3.6, log_2_ = 1.85) and histone H2A.Z (FC = 2.6, log_2_ = 1.38) (Fig 6).

## Discussion

In the present study several findings could be achieved. First of all, elevated IOP due to thermic cauterization could mimic the effect of glaucomatous course of disease, which could be shown mainly through apoptosis events in the retinal ganglion cell layer, and confirmed by retinal nerve fiber layer and optic nerve investigations. In addition, dynamic relations of quantitative proteomic expression pattern could be analyzed, revealing distinct shifting of α- and β-crystallins as a reaction of varying progression with respect to the disease state. Lastly, inversion of the β-crystallin B2 condition within the retina at early disease state effectuated clear neuroprotective effects, which resulted in a reduction of neuronal impairment in the course of elevation of intraocular pressure. The data suggest that crystallin proteins might feature as key molecules when it comes to glaucoma, with potential in diagnostics as well as in medical treatment.

Crystallins comprising of alpha, beta and gamma crystallins have gone within a relatively short period of time from being considered lens-specific proteins to being recognized as well as neuronal and RGC proteins [25, 26]. Specific regulations of HSP’s and HspB’s have been observed in context with several neurodegenerative diseases [27, 28]. According to this, crystallins have been seen to be temporarily differently expressed besides the brain as well within the rat retina after various forms of injury [34, 40], indicating their involvement in injury and postinjury repair. Several studies could already show that higher levels of HspB5 seem to be connected to a variety of neurodegenerative disorders like Alzheimer’s disease and Parkinson’s disease [41, 42]. Especially several experimental studies indicated a neuroprotective potential of β-crystallin B2 when it comes to neuronal impairment [30–32].

In context with regulation of crystallins, several findings were recently reported on mRNA and protein levels in both hereditary and experimental models of glaucoma [34, 43, 44] and HspB5 expression was found to be stimulated in glaucomatous optic nerves of primates [45].

In our study, dynamic relations of quantitative proteomic expression pattern revealed distinct shifting of α- and β-crystallins as a reaction of varying progression with respect to the disease state, showing a unique expression characteristic. While all α- and β-crystallins were found down-regulated after three weeks of IOP elevation, a distinct up-regulation after seven weeks of elevated IOP could be seen. Comparable findings at mRNA level could be already shown by Piri et al. [34]. Interestingly, crystallin expression pattern shifts due to the duration of elevated IOP, showing down-regulation of crystallins at mRNA levels 2 weeks and up-regulation to control levels 5 weeks after IOP elevation. γ-crystallin B and γ-crystallin C, however, didn’t show this opposite trend, but progressive rise of the relative expression state with continuous IOP elevation. It is suggested that crystallin transcription might be stimulated throughout the RGC degeneration in response to increased levels of IOP or as a response to the IOP elevation independently from the RGC degeneration [34].

While the intense down-regulation of the crystallin protein family remains poorly understood and is most likely connected to sudden increase of intraocular pressure, we propse that the up-regulation is a delayed cellular protection mechanism. The injection of β-crystallin B2 seems to confirm this prediction. In our study injection of β-crystallin B2 had a clear neuroprotective effect on RGCs. Protein application with starting of IOP rise reverses the condition of β-crystallin B2 on the molecular level, resulting in tremendous neuroprotective effects for RGCs.

According to these findings, several lines of evidence indicate that application of β-crystallins to injured retinal tissue transforms RGCs into a robust regenerative state, enabling them to regrow axons at higher growth rates [46, 47]. Addition of purified crystallin isoforms enhances axonal growth in animal models *in vitro* [26] and *in vivo* [25], thereby possibly involving either inflammatory events and macrophage activation or non-inflammatory effects by mediating through ciliary-neurotrophic factor (CNTF) [25]. Moreover, it has been seen that β-crystallin B2 (crybb2) and β-crystallin B3 (crybb3) expression is upregulated and released into the medium by regenerating retinal tissue *in vitro*. Crystallin expression in filopodial processes of RGCs and their axons leads to the suggestion that crystallins may play a crucial role in axogenesis and growth cone formation [26].

As members of the small heat shock protein family, HspB proteins possess of chaperone-like capabilities and incremental expression as a reaction to stress could be observed in different cells and tissues [48, 49]. These findings indicate that crystallins themselves belong directly to the cellular defense mechanism. With respect to function and mode of action of α-crystallins promising insights could be gained in recent years [49–52], but for β-crystallins not many interaction partners are known so far.

**Fig 7:**
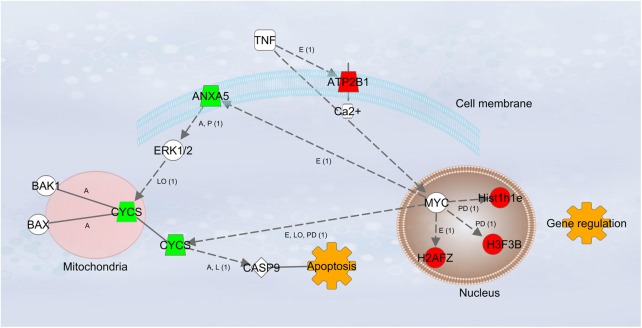
Molecular interaction network by Ingenuity Pathway Analysis. Down-regulation in green, up-regulation marked in red. A = Activation, LO = Localization, E = Expression, PD = Protein/DNA interaction, L = molecular cleavage, P = Phosphorylation. ANXA5 = Annexin V, CYCS = Cytochrome c, somatic, ATPB1 = Calcium-transporting ATPase 1, Hist1h1e = Histone H1, H3F3B = Histone H3, H2AFZ = Histone H2A.Z, MYC = myc proto-oncogen

The mechanism by which β-crystallin B2 exerts its neuroprotective function remains obscure, but a connection to a chaperone function might be possible. Certainly, other groups claim that it might act via CNTF [25]. By comparing mass-spectrometric LFQ intensities from crystallin injected eyes to IOP treated animals, we found clear evidence for shifts in calcium dependent signaling, like annexin A5 and calcium-transporting ATPase 1. Annexin A5, which was found down-regulated with a fold-change of 0.053 in crystallin animals, is not only a marker for apoptosis [53], but is cross-linked to the respective cellular calcium level. Annexin A5 is connected to cytochrome C via the mitogen-activated protein kinase 1 [54]. Cytochrome C, which is released from mitochondria, depending on the calcium level, is known as a direct driver for apoptosis by activating caspase 9 [55]. Down-regulation of somatic cytochrome C through crystallin injection might be a reason for the lower RGC apoptosis rate (Fig 7).

Furthermore, we found an up-regulation of three histone proteins (histone H1, H3 and H2A.Z), located in the nucleus. The transcription factor myc proto-oncogene might be stimulated by annexin A5 and seems to be directly connected to the expression of the histone proteins. Histone molecules form nucleosomes with the DNA and are therefore directly involved in DNA repair, gene expression- and regulation.

It remains uncertain which upstream molecules are directly activated by crystallin proteins and which retinal cells are mainly involved in the regulation of their protective features, although our study could show that β-crystallin B2 seems to be predominantly uptaken by the RGCs. However, the here presented downstream reactions illustrate a novel mode of action, which could underlie the promising effects of β-crystallins.

Due to the relatively small size and stability of crystallin proteins, one could also speculate that crystallin molecules are involved in secretion and processes of cellular re-uptake. There are different studies, which could proof secretion of crystallins into the aqueous- and vitreous humor as well as cellular secretion events with respect to exosomes vesicle transportation [56, 57].

Concluding, we found a typical pattern of retinal crystallin expression during the course of the disease on proteomic levels in an experimental animal glaucoma model serving as key molecules and potentially specific biomarkers. Besides this we found neuroprotective function of β-crystallin B2 in reversing the time depending natural course of the disease. We were able to confirm the neuroprotective character of β-crystallin B2 and revealed some calcium depended proteins, which might be responsible for increased RGC survival. Furthermore, we suggest the protein family of crystallins in general to play a major role in relation of glaucoma, which was indicated by the distinct response in crystallin expression levels due to IOP elevation. Nevertheless, continuing more in-depth investigations will be necessary to confirm these results and to explore the potential role of β-crystallin B2 in terms of neuronal regeneration. Especially the identification of direct interaction partners of β-crystallin B2 represents a key role in this context.
